# Improvement of Initial Azimuth Estimation Time for a North-Finding System Using Low-Cost MEMS Sensors and a Compact 3-Axis Turntable in Challenging Environments

**DOI:** 10.3390/s26165313

**Published:** 2026-08-21

**Authors:** Taisei Hayashi, Daisuke Terada

**Affiliations:** Department of Mechanical Systems Engineering, National Defense Academy of Japan, Hashirimizu 1-10-20, Yokosuka 239-8686, Kanagawa, Japan

**Keywords:** north finding, low-cost sensor, initial azimuth estimation, due north detection

## Abstract

This paper proposes a method for reducing the initial azimuth estimation time of a north-finding system employing low-cost sensors and a compact 3-axis turntable. The system is capable of operating in non-horizontal environments, magnetically disturbed environments, and environments where Global Navigation Satellite System (GNSS) signals are unavailable. Detection of due north without prior azimuth information was evaluated through indoor experiments under the aforementioned conditions. During each rotation, the compact 3-axis turntable was kept horizontal and the acceleration and angular velocity were measured in 16 directions at 22.5° intervals. By including the final position coinciding with the initial one, a total of 17 measurement points were obtained per lap. This process was repeated for 77 laps. For statistical evaluation, 5000 bootstrap replications were generated. Detection of due north was then performed using these datasets and the relationship between the number of laps and the estimation error was statistically analyzed. Consequently, it was confirmed that the root mean square (RMS) error becomes less than 1° after ten laps, corresponding to a data acquisition time of approximately 1.3 h. Compared to our previous study, the required estimation time is reduced by approximately 2 h.

## 1. Introduction

A gyrocompass is commonly used to determine azimuth in environments with magnetic interference [1]. However, they are large, expensive, and difficult to maintain, prompting recent interest in methodologies employing Micro Electro-Mechanical Systems (MEMS) gyro sensors or Fiber-Optic Gyroscopes (FOG) to estimate azimuth. To estimate azimuth employing a MEMS gyro sensor, it is first necessary to estimate the initial azimuth the angle between the sensor’s azimuth and due north (initial azimuth). There are two types of due north detection employing MEMS gyro sensors: one estimates the azimuth while the sensor rotates continuously, and the other involves holding the sensor stationary in multiple orientations to estimate the angle between the sensor azimuth and due north. Zhang et al. [2] proposed a method for determining north employing a MEMS gyro sensor and a MEMS accelerometer based on the rotation modulation technique. Luo et al. [3] measured angular velocity in 72 directions to estimate the angle between the sensor azimuth and due north. To estimate azimuth with an FOG, observation of the slow gravity drift in the inertial space allows one to determine the Earth axis [4]. On the other hand, for small vehicles or systems involving cooperative control of multiple small vehicles, these devices need to be constructed with low-cost and compact components. Against this background, we focus on a method for detecting due north employing low-cost MEMS gyro sensors. In our previous study [5], the authors developed a portable north-finding system for use in non-horizontal or magnetically disturbed environments or in locations where Global Navigation Satellite System (GNSS) signals are unavailable. The system was designed to be mountable on small vehicles. The hardware platform consists of low-cost MEMS sensors and a compact 3-axis turntable. Following the approach proposed by Luo et al. [3], measurements were carried out at 16 uniformly spaced directions, and due north was determined through phase estimation.

However, our previous study [5] encountered four challenges. First, in estimating the accelerometer offset error and scale error, our previous study employed an acceleration point cloud to estimate these parameters. However, acquiring the acceleration point cloud before every lap was time-consuming. To address this challenge, this study models the offset and scale errors as linear functions of temperature from a separately obtained acceleration point cloud data. This reduces the measurement time by eliminating the need to estimate these errors before every lap. Next, the measurement time required for due north detection was approximately 3.3 h, which poses a practical limitation. To overcome this limitation, the following improvements are introduced in this study:

- The measurement duration in each direction is increased from 7.5 s to 25 s.
- For attitude estimation, this study employs a quaternion-based method. Because the acceleration vector after offset and scale error compensation theoretically lie on a sphere with a radius equal to the gravitational acceleration g, the quaternion is computed from the dot product and cross-product of the normalized acceleration vector and a reference attitude vector. The attitude is then estimated using the computed quaternion.
- In the weighted averaging process, this study utilizes the angular velocity and acceleration along the *x*- and *y*-axes to estimate parameters. Furthermore, detected outliers are treated as missing data, reducing the influence of potentially unreliable measurements.

In the previous study, verification of the rotational accuracy about the *z*-axis in the compact 3-axis turntable was limited to a visual comparison of two images. To address this challenge, image differencing is employed to evaluate the rotational accuracy more quantitatively. Finally, in the process of verifying the number of laps required for the RMS error of due north detection to fall below 1° (hereinafter referred to as the “multiple lap trials”), our previous study evaluated combinations of sets of each cycle to verify the results using sampling without replacement. However, the statistical uncertainty of the results was not evaluated. To address this limitation, in this study we generate 5000 bootstrap replications for each direction. Using these bootstrap replications, we investigate the number of laps required to achieve an RMS error of less than 1° after multiple lap trials conducted through 10,000 samplings with replacement for each direction.

The rest of this paper is structured as follows: Section 1 describes the background and objectives; Section 2 denotes the coordinate systems; Section 3 provides a detailed explanation of the signal processing methods; Section 4 presents the verification of our compact 3-axis turntable and discusses the results; Section 5 described and analyzes the experimental results; finally, Section 6 summarizes the obtained findings and concludes the paper. The Appendix A describes the method of signal processing from our previous study [5].

## 2. The Compact 3-Axis Turntable and Its Coordinate System

**Figure 1:**
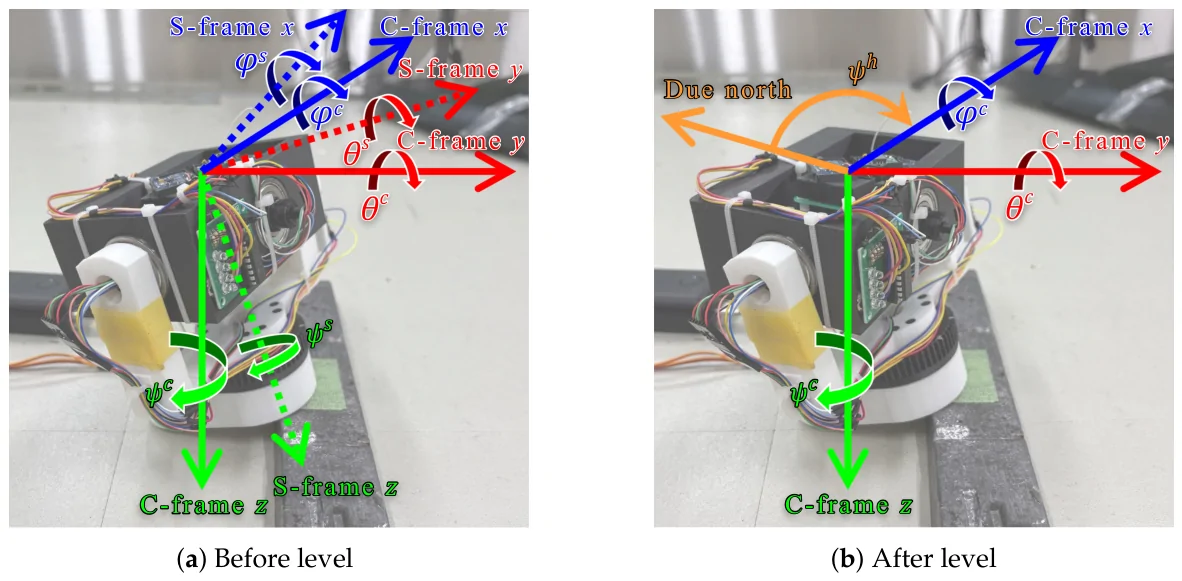
Overview of compact 3-axis turntable and its coordinate system.

An overview of the compact 3-axis turntable and its coordinate system is shown in Figure 1. The coordinate system is right-handed with the *z*-axis pointing downward. The blue, red, and green arrows shown in the figure correspond to the *x*-, *y*-, and *z*-axes, respectively. Solid lines and dashed lines respectively represent the computer frame (C-frame), which is a rotation relative coordinate system, and the sensor (local) coordinate system (S-frame). The S-frame denotes the coordinate frame fixed to the sensor body, while the C-frame represents the reference coordinate system used for rotational transformations. The attitude of the C-frame is represented using a quaternion. For the rotation convention, the blue, red, and green curved arrows denote rotations around the *x*-, *y*-, and *z*-axes of the C-frame, referred to as C-frame roll ($\phi^{c}$), C-frame pitch ($\theta^{c}$) and C-frame yaw ($\psi^{c}$), respectively. In addition, rotations around the *x*-, *y*-, and *z*-axes of the S-frame are referred to as S-frame roll ($\phi^{s}$), S-frame pitch ($\theta^{s}$), and S-frame yaw ($\psi^{s}$), respectively. The straight and curved orange arrows show the direction of the due north and the heading angle ($\psi^{h}$), respectively. Note that the compact 3-axis turntable employed in this study is identical to that used in our previous study [5].

## 3. Signal Processing

### 3.1. Overview

**Figure 2:**
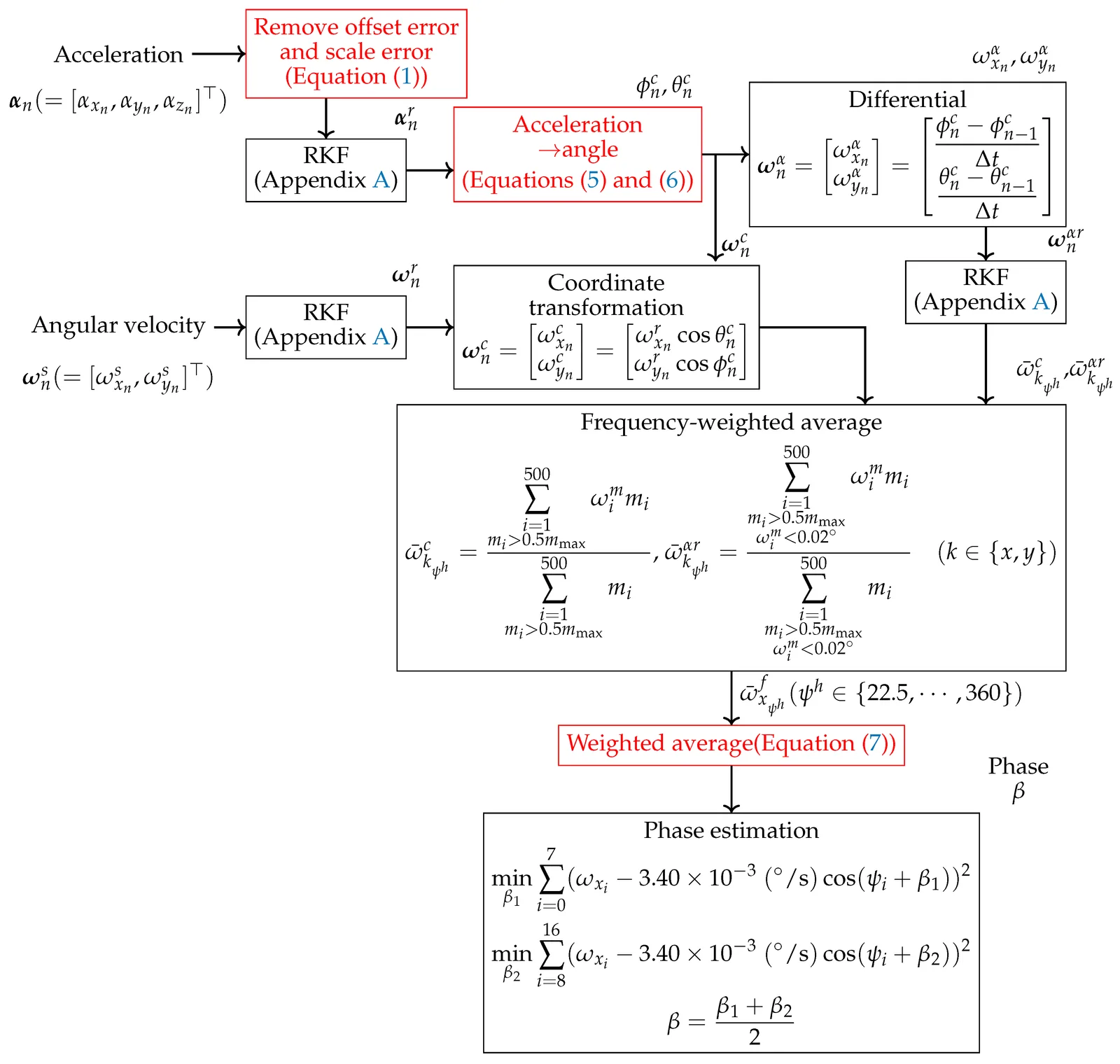
Process flow of the proposed system.

The signal processing flow is shown in Figure 2. In the figure, the boxes represent signal processing. The black boxes show where the process is the same as our previous study [5], while the red boxes show where the process is changed in this study. The signal processing of our previous study [5] is written in the Appendix A in detail. The offset error and scale error are removed from the accelerometer signals $\alpha_{n}$ using Equation (1). The details of Equation (1) are described in Section 3.2. The acceleration signal, which removes the offset error and the scale error, has outliers. To remove these outliers, we utilize the Robust Kalman Filter (RKF) [6]. Details of the RKF are described in Appendix A. The processed signals $\alpha_{n}^{r}$ are normalized by the l2 norm. Utilizing the normalized acceleration vector and a reference vector, the quaternion is calculated by the dot product and the cross-product. This quaternion is then used to transform the sensor data into the horizontal coordinate system of the C-frame. The quaternion is subsequently converted into Euler angles using Equations (5) and (6). The details of Equations (5) and (6) are described in Section 3.3. These angles are then converted to $\omega_{n}^{\alpha }$ through numerical differentiation. The angular velocity $\omega_{n}^{\alpha r}$ estimated from the accelerometer is the consequence of processing $\omega_{n}^{\alpha }$ with the RKF to remove outliers. The angular velocity $\omega_{n}^{s}$ measured from the gyro sensor is also processed using the RKF to remove outliers. The same RKF algorithm is used to remove outliers from the signals measured by the gyro and acceleration sensors; however, the state, observations, system noise, and coefficients are different. Details are provided in Appendix A. The processed angular velocity $\omega_{n}^{r}$ is converted to the C-frame. The frequency-weighted averages of the angular velocity obtained from the gyro sensor and accelerometer are ${\bar{\omega }}_{k_{\psi^{h}}}^{c}$ and ${\bar{\omega }}_{k_{\psi^{h}}}^{\alpha r}$, respectively, which are calculated utilizing the frequency distribution of $\omega_{n}^{c}$ and $\omega_{n}^{\alpha r}$. Then, the weighted average value ${\bar{\omega }}_{x_{\psi^{h}}}^{f}$ is estimated using Equation (7). The details of Equation (7) are described in Section 3.4. Finally, the β phase is estimated based on the nonlinear least squares method [7]. This study utilized “curve_fit” function in SciPy (version 1.18.0) Python (version 3.14.6) library.

### 3.2. Removing the Offset Error and Acceleration Scale Error

Let the signal from the accelerometer $\alpha_{n}^{s}\left(={\left[\alpha_{x_{n}}^{s},\alpha_{y_{n}}^{s},\alpha_{z_{n}}^{s}\right]}^{⊤}\right)$, the offset error of the acceleration $\alpha_{n}^{c}\left(={\left[\alpha_{x_{n}}^{c},\alpha_{y_{n}}^{c},\alpha_{z_{n}}^{c}\right]}^{⊤}\right)$, the scale error of the acceleration $\alpha_{n}^{r}\left(={\left[\alpha_{x_{n}}^{r},\alpha_{y_{n}}^{r},\alpha_{z_{n}}^{r}\right]}^{⊤}\right)$, and the processed signal $\alpha_{n}\left(={\left[\alpha_{x_{n}},\alpha_{y_{n}},\alpha_{z_{n}}\right]}^{⊤}\right)$. The offset error and scale error of the acceleration can be removed by the following Equation (1) [8].

(1) $$\alpha_{n}=\left[\begin{array}{c} \alpha_{x_{n}}\\ \alpha_{y_{n}}\\ \alpha_{z_{n}} \end{array}\right]=\left[\begin{array}{c} \left(\alpha_{x_{n}}^{s}-\alpha_{x}^{c}\right)/\alpha_{x}^{r}\\ \left(\alpha_{y_{n}}^{s}-\alpha_{y}^{c}\right)/\alpha_{y}^{r}\\ \left(\alpha_{z_{n}}^{s}-\alpha_{z}^{c}\right)/\alpha_{z}^{r} \end{array}\right]$$

**Figure 3:**
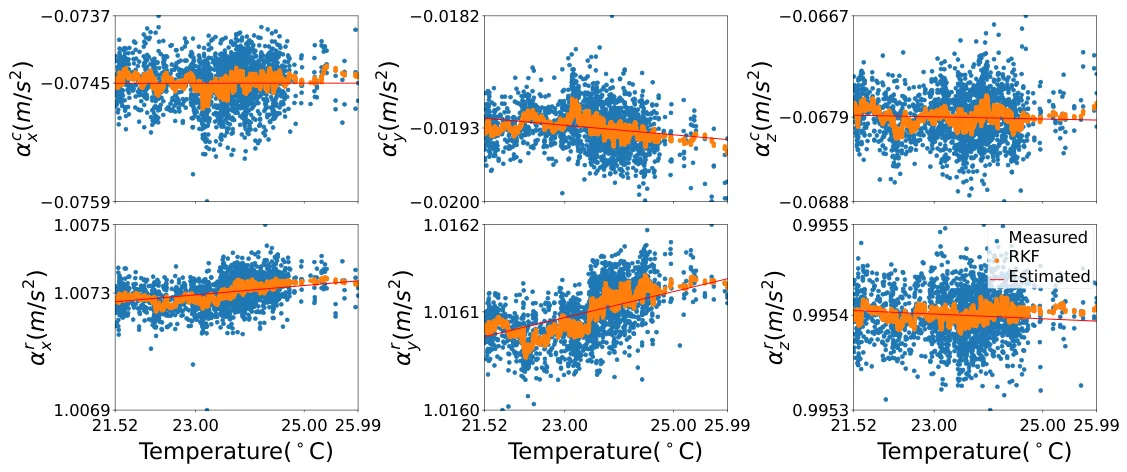
The offset error $\alpha_{n}^{c}$ and scale error $\alpha_{n}^{r}$ under different temperature environments.

The offset error $\alpha_{n}^{c}$ and scale error $\alpha_{n}^{r}$ are estimated by the following steps. First, an acceleration point cloud in three-dimensional (3D) space is generated by rotating the compact 3-axis turntable in increments of 28.1° about the *x*- and *y*-axes and stopping at each position. Next, since this point cloud contains errors, the triaxial ellipsoid is estimated utilizing these data via the least squares method. The estimated center and radius of the triaxial ellipsoid become the offset error $\alpha_{n}^{c}$ and scale error $\alpha_{n}^{r}$, respectively. It is known that the offset error $\alpha_{n}^{c}$ and scale error $\alpha_{n}^{r}$ of the accelerometer are correlated with temperature. Therefore, the triaxial ellipsoid was estimated from 2072 trials under different temperature conditions between 21.52 °C and 25.99 °C. The RKF is used to remove outliers from the estimated values. The least squares method is used to estimate the linear function relating to temperature. The estimation results for the offset error $\alpha_{n}^{c}$ and scale error $\alpha_{n}^{r}$ under different temperature environments are shown in Figure 3. The figures plot the offset error or scale error on the vertical axis and the temperature on the horizontal axis. The blue circles, orange circles, and red lines represent the estimated values, values after outlier correction utilizing the RKF, and regression line, respectively. The estimated parameters of the linear function are shown in Table 1.

### 3.3. Quaternion Estimation Using the Acceleration Vector and Reference Attitude Vector

The quaternion qnAB representing the rotation from vector A to vector B is expressed by the following Equations (2)–(4) [9]:

(2) qnAB=q0nABq1nABq2nABq3nAB=cos(mn/2)rxnsin(mn/2)rynsin(mn/2)rznsin(mn/2)

(3) $$r_{n}={\left[r_{x_{n}},r_{y_{n}},r_{z_{n}}\right]}^{⊤}=\alpha_{n}\times \alpha_{0}$$

(4) $$m_{n}=\alpha_{n}\cdot \alpha_{0}$$

where $r_{n}$ is the cross-product of $\alpha_{n}$ and $\alpha_{0}$, while $m_{n}$ is the dot product of $\alpha_{n}$ and $\alpha_{0}$. Let vector A be normalized acceleration $\alpha_{n}$ with the offset error and scale error removed and let vector B be the normalized acceleration $\alpha_{0}\left(={\left[0,0,-1\right]}^{⊤}\right)$ at the attitude ${\left[\phi^{c},\theta^{c},\psi^{c}\right]}^{⊤}={\left[0,0,0\right]}^{⊤}$. Then, these relationships are always relative and equivalent to the attitude of the translational coordinate system. The quaternion corresponding to the acceleration signal $\alpha_{n}$ is expressed by the following Equation (5):

(5) $$q_{n}^{\alpha }=\left[\begin{array}{c} q_{0_{n}}\\ q_{1_{n}}\\ q_{2_{n}}\\ q_{3_{n}} \end{array}\right]=\left[\begin{array}{c} \cos(m_{n}/2)\\ r_{x_{n}}\sin\left(m_{n}/2\right)\\ r_{y_{n}}\sin\left(m_{n}/2\right)\\ r_{z_{n}}\sin\left(m_{n}/2\right) \end{array}\right]$$

and the quaternion is converted into Euler angles by the following equation.

(6) $$\left[\begin{array}{c} \phi_{n}^{c}\\ \theta_{n}^{c} \end{array}\right]=\left[\begin{array}{c} \tan^{-1}\frac{q_{3_{n}}^{2}-q_{2_{n}}^{2}-q_{1_{n}}^{2}+q_{0_{n}}^{2}}{2(q_{0_{n}}q_{1_{n}}+q_{2_{n}}q_{3_{n}})}\\ \sin^{-1}\left\{2\left(q_{1_{n}}q_{3_{n}}-q_{0_{n}}q_{2_{n}}\right)\right\} \end{array}\right]$$

### 3.4. Weighted Average

The frequency-weighted averages ${\bar{\omega }}_{k_{\psi^{h}}}^{c}\;\left(k\in \left\{x,y\right\}\right)$ and ${\bar{\omega }}_{k_{\psi^{h}}}^{\alpha r}\;\left(k\in \left\{x,y\right\}\right)$ of the angular velocity are obtained from the gyro sensor and the accelerometer, respectively. Both are computed following the procedure described in our previous study. Then, the weighted average of the angular velocity ${\bar{\omega }}_{x_{\psi^{h}}}^{f}$ is expressed by the following equation:

(7) $$\begin{array}{c} {\bar{\omega }}_{x_{\psi^{h}}}^{f}=\left\{\begin{array}{cc} a_{0}{\bar{\omega }}_{x_{\psi^{h}}}^{c}+a_{1}{\bar{\omega }}_{x_{\psi^{h}}}^{\alpha r}+a_{2}{\bar{\omega }}_{y_{\psi^{h}}}^{c}+a_{3}{\bar{\omega }}_{y_{\psi^{h}}}^{\alpha r}\;\; & \mathrm{if}\;\begin{array}{c} -0.005^{\circ }\leq {\bar{\omega }}_{x_{\psi^{h}}}^{c}\leq 0.005^{\circ }\\ \wedge -0.005^{\circ }\leq {\bar{\omega }}_{x_{\psi^{h}}}^{\alpha r}\leq 0.005^{\circ }\\ \wedge -0.005^{\circ }\leq {\bar{\omega }}_{y_{\psi^{h}}}^{c}\leq 0.005^{\circ }\\ \wedge -0.005^{\circ }\leq {\bar{\omega }}_{y_{\psi^{h}}}^{\alpha r}\leq 0.005^{\circ } \end{array}\\ \mathrm{Missing} & \mathrm{otherwise} \end{array}\right. \end{array}$$

where a is a parameter vector given by a=[0.6,0.4,0.0,0.0].

**Figure 4:**
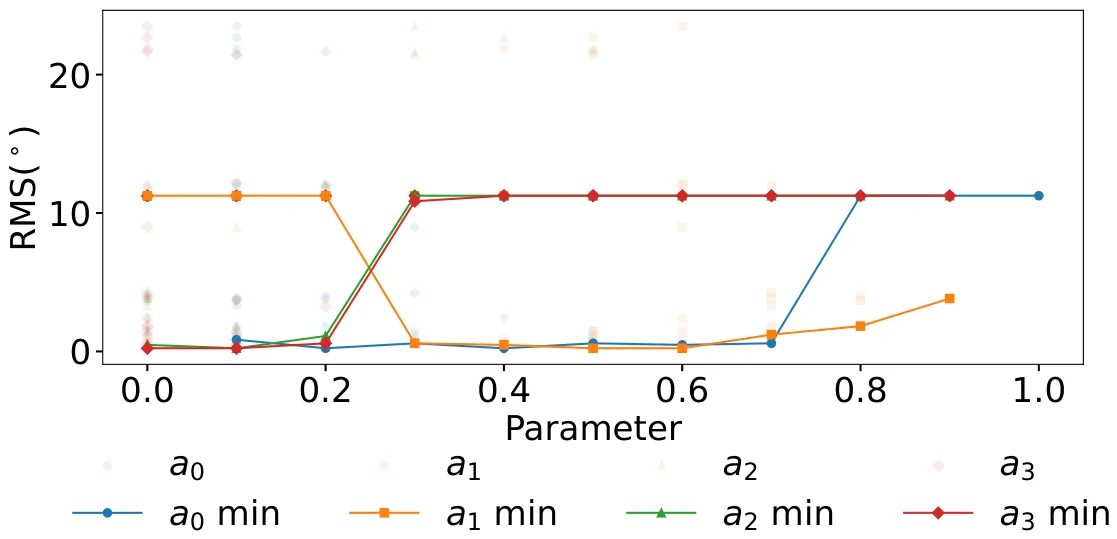
Parameter estimation results for a.

The parameter vector is estimated based on the lowest RMS error utilizing 77 laps data measured in the experiment. The estimation results for the parameter vector a are shown in Figure 4. The figure plots the parameter values on the horizontal axis and the RMS error on the vertical axis. The blue, orange, green, and red lines in the figure represent parameters $a_{0}$ through $a_{3}$, respectively, and the minimum value is indicated by a line. From the figure, the RMS error for parameter $a_{0}$ of ${\bar{\omega }}_{x_{\psi^{h}}}^{c}$ is low in the range from 0.1 to 0.7. The RMS error for parameter $a_{1}$ of ${\bar{\omega }}_{x_{\psi^{h}}}^{\alpha r}$ is low in the range from 0.3 to 0.6. For ${\bar{\omega }}_{y_{\psi^{h}}}^{c}$ and ${\bar{\omega }}_{y_{\psi^{h}}}^{\alpha r}$, parameters $a_{2}$ and $a_{3}$ yield low RMS errors in the range from 0.0 to 0.2. In other words, to detect north it is sufficient to use ${\bar{\omega }}_{x_{\psi^{h}}}^{c}$ and ${\bar{\omega }}_{x_{\psi^{h}}}^{\alpha r}$.

### 3.5. Resampling Utilizing the Nonparametric Bootstrap Method

Detection of due north is carried out using measurement datasets, after which the relationship between the number of laps and the estimation error is statistically analyzed. For statistical evaluation, 5000 bootstrap replications were generated using a nonparametric bootstrap method with resampling with replacement [10]. According to Efron [11], 1000 bootstrap replications are generally sufficient for bootstrap confidence interval estimation. However, to obtain more stable and robust estimates, 5000 bootstrap replications were generated. The compact 3-axis turntable measures every 22.5° from the starting point, completes one lap, and then performs measurement again at the starting point, obtaining data for a total of 17 directions. The samples estimated by the compact 3-axis turntable are $X_{\left(17,77\right)}\left(=\left[X_{0,0}\cdots X_{0,76}\right]\cdots \left[X_{16,0}\cdots X_{16,76}\right]\right)$ and the bootstrap replications generated by the nonparametric bootstrap method $X_{\left(17,5000\right)}^{*}\left(=\left[X_{0,0}^{*}\cdots X_{0,4999}^{*}\right]\cdots \left[X_{16,0}^{*}\cdots X_{16,4999}^{*}\right]\right)$. Under this assumption, resampling by the nonparametric bootstrap method is shown in the following Algorithm 1.
**Algorithm 1** Resampling Utilizing the Nonparametric Bootstrap Method**Require:** 
$X(=\left[X_{0,0}\cdots X_{0,76}\right]\cdots \left[X_{16,0}\cdots X_{16,76}\right])$**Ensure:** 
$X^{*}\left(=\left[X_{0,0}^{*}\cdots X_{0,4999}^{*}\right]\ldots \left[X_{16,0}^{*}\ldots X_{16,4999}^{*}\right]\right)$  1:**for** k←1**to** 17 **do**  2:      **for** h←1 **to** 5000 **do**  3:            **for** i←1 **to** 77 **do**  4:                  i←∼U(0,76)  5:                  $S_{h}\leftarrow X\left[k,i\right]$  6:            **end for**  7:            $X^{*}\left[k,h\right]\leftarrow M$edian(***S***)
  8:      **end for**  9:**end for**10:**return**$\;X^{*}$

In the above algorithm, *i* is determined utilizing the “random.integers” function [12] in the NumPy (version 2.4.6) Python (version 3.14.6) library, with random numbers generated using PCG64 and an initial seed of 9. Employing bootstrap replications $X^{*}$, multiple lap trials were conducted through 10,000 samplings with replacement, and the number of laps required to achieve an RMS error of less than 1° was investigated. The number of resampling trials was determined considering the computational resources available.

## 4. Verification of Accuracy of the Compact 3-Axis Turntable S-Frame Yaw ($\psi^{s}$) Motion

**Figure 5:**
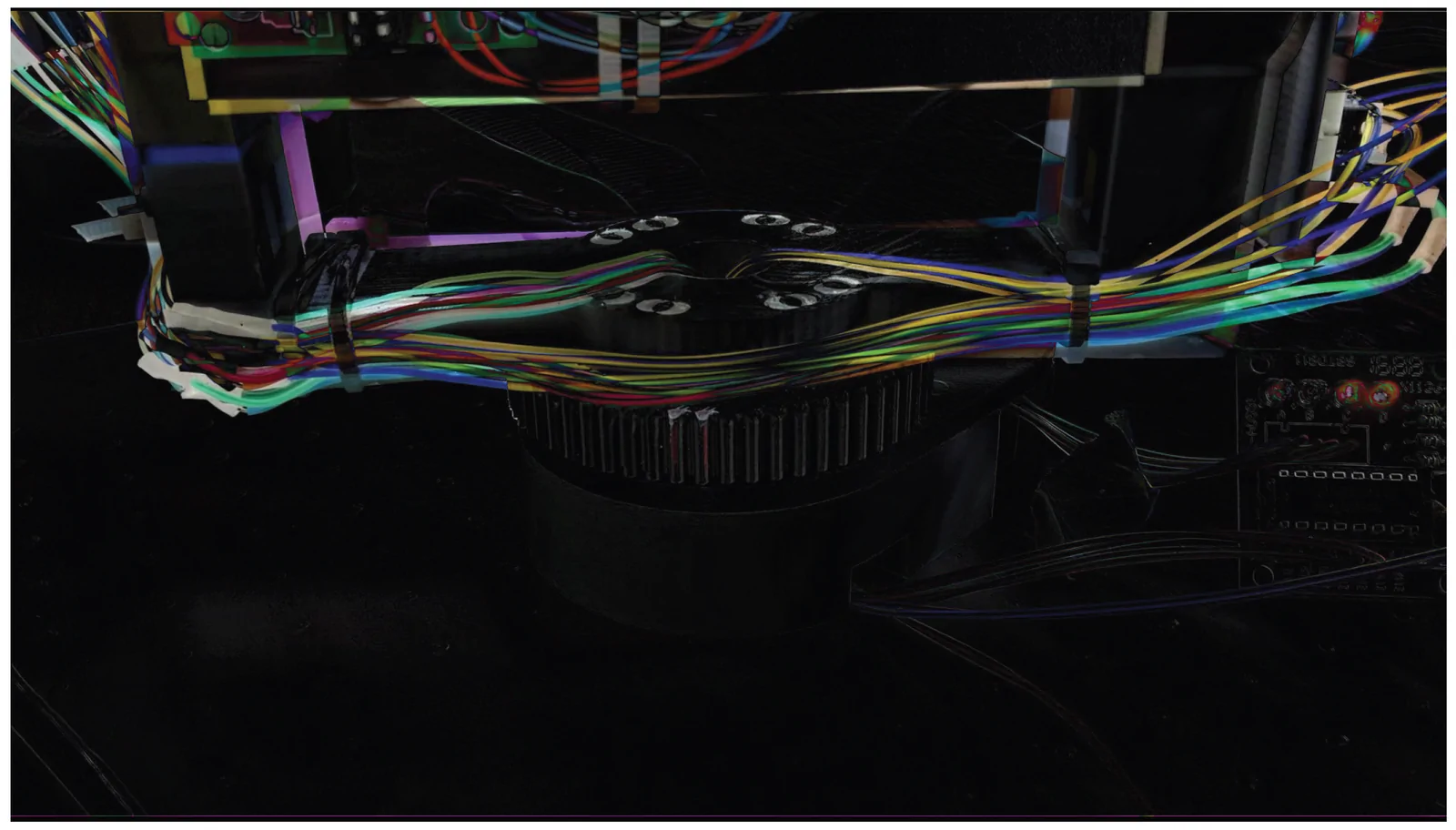
The initial image and an image intentionally rotated about the S-frame yaw ($\psi^{s}$), then combined using difference compositing.

**Figure 6:**
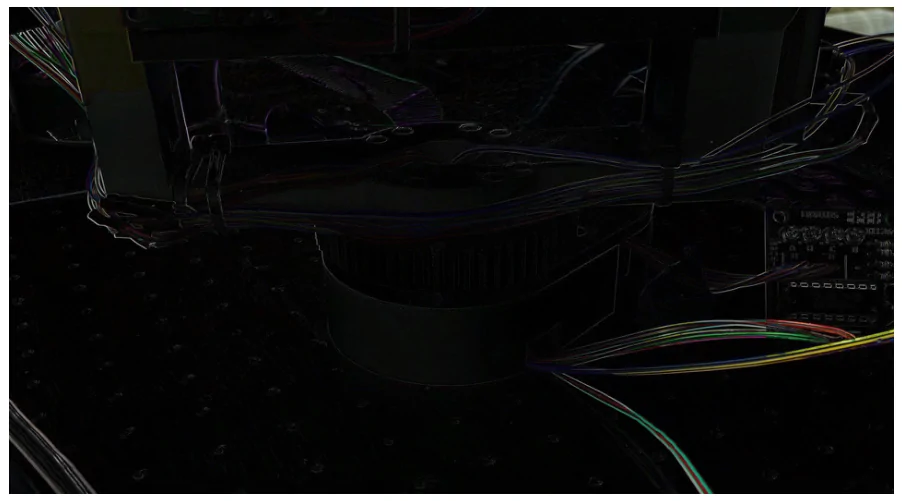
Before and after start images combined by difference compositing.

To evaluate the accuracy of the compact 3-axis turntable in the S-frame yaw ($\psi^{s}$), the compact 3-axis turntable was repeatedly commanded to rotate by 22.5° about the S-frame yaw ($\psi^{s}$) until 100 complete laps had been performed. The images captured before and after these 100 laps were used for image-based analysis. The verification was performed employing image editing software (Adobe Photoshop (version 26.11.3)) with difference compositing (difference mode). Difference mode works according to the color information in each picture and subtracts either the blend color from the base color [13]. Therefore, if the alignment marks are in the same location before and after the start, the image will be displayed in black. Figure 5 shows the result of difference compositing between the initial image and an image intentionally rotated about the S-frame yaw ($\psi^{s}$) The figure shows that the alignment markers are located at different positions. Figure 6 shows the difference-composited image generated from the images captured before and after the experiment. The figure shows that the alignment markers remained nearly unchanged in position, indicating that the compact 3-axis turntable achieved S-frame yaw ($\psi^{s}$) rotations consistent with the control signals.

## 5. Experimental Results and Discussions

**Figure 7:**
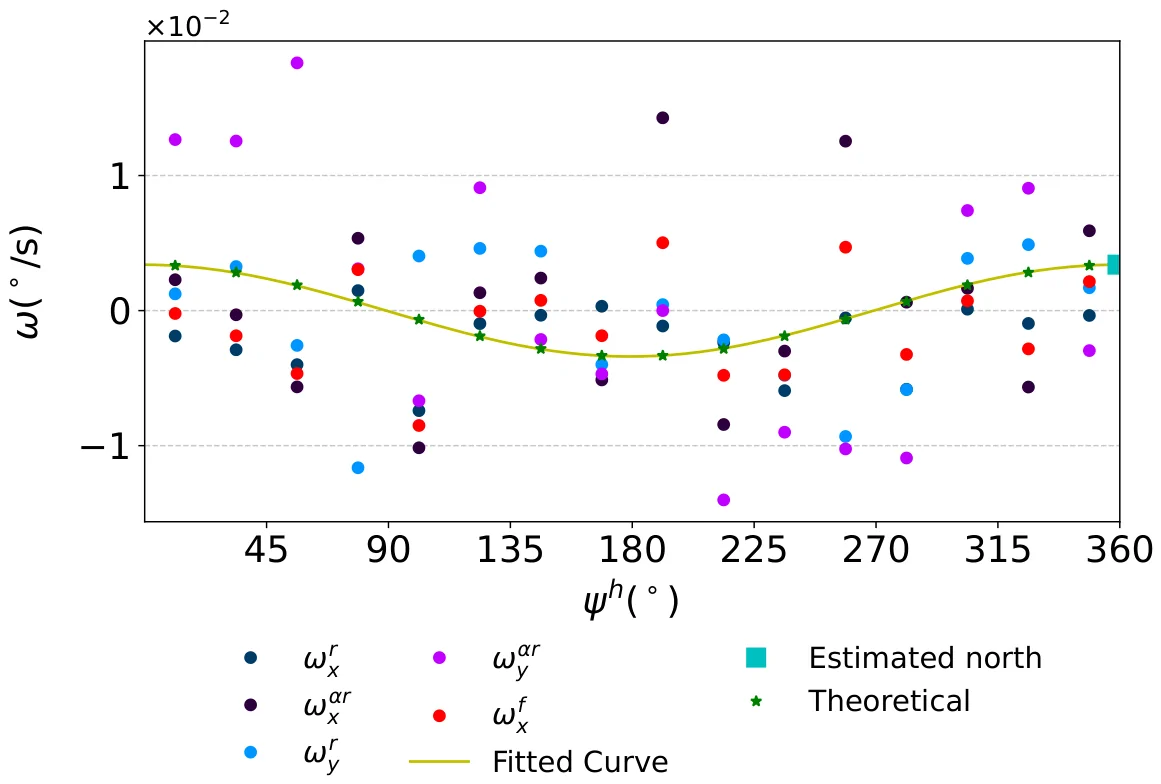
An example of the results obtained during a single lap.

To evaluate the proposed method, an experiment was performed in Education and Research Building A at the National Defense Academy of Japan. Before the experiment, the following steps were performed. First, the azimuth of a reference wall in the experimental area was calculated using an online azimuth calculation tool provided by the Geospatial Information Authority of Japan (GSI) [14]. Then, the compact 3-axis turntable was aligned with the reference wall by adjusting the S-frame yaw ($\psi^{s}$). Finally, the device was rotated to an initial heading of $\psi^{h}$ = 101.25° to verify a situation in which due north was not known. The compact 3-axis turntable was used to keep the sensor horizontal before each direction measurement. Note that since the experiments were conducted in a non-horizontal environment, due north can be estimated utilizing phase estimation based on the confirmed accuracy of the S-frame yaw ($\psi^{s}$). The compact 3-axis turntable was rotated about the S-frame yaw ($\psi^{s}$) at intervals of $22.5^{\circ }$, and data were obtained uniformly at the same 16 points per lap. An example of the results obtained during a single lap is shown in Figure 7. In the figure, the vertical axis represents the angular velocity and the horizontal axis represents the heading angle. The blue, purple, and red dots, yellow lines, light blue square mark, and green dots show average values of $\omega_{x}^{c}$, $\omega_{x}^{\alpha r}$, and $\omega_{x}^{f}$ for the estimated cosine curve, estimated due north, and theoretical values, respectively. This example demonstrates successful due north detection.

However, due north could not be estimated in all trials. Therefore, the number of laps required to estimate due north with an RMS error of less than 1° needed to be verified. Employing 5000 laps of data generated by the nonparametric bootstrap method described in Section 3.5, multiple lap trials were conducted by performing 10,000 samplings with replacement. A total of 10,000 samplings with replacement were generated utilizing the “random.choice” function [12] in the NumPy (version 2.4.6) Python (version 3.14.6) library, with the procedure repeated ten times employing different initial seeds ranging from 0 to 9.

**Figure 8:**
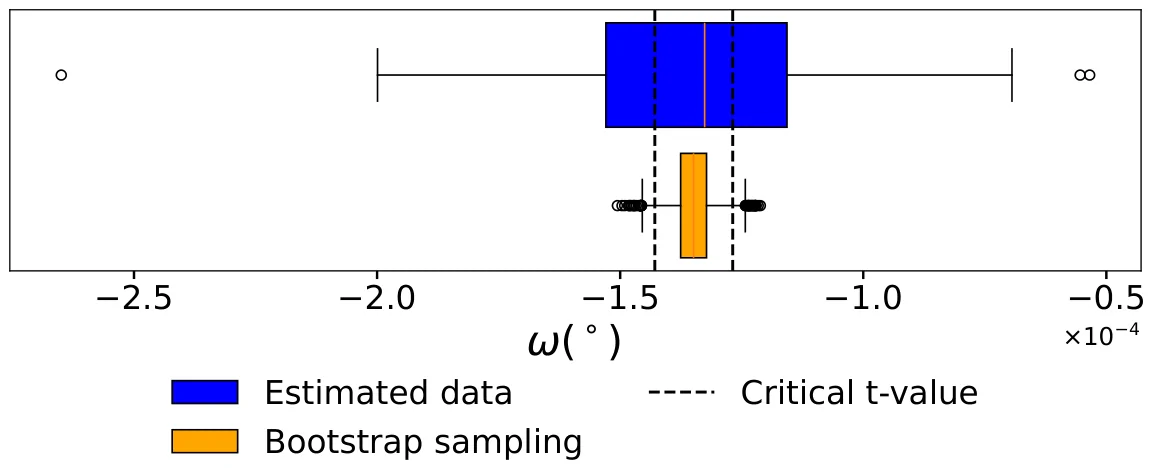
Samples estimated by the proposed method using the frequency-weighted average angular velocity obtained from the gyro sensor *x*-axis at $\psi^{h}=90^{\circ }$ (${\bar{\omega }}_{x_{90}}^{c}$) compared to resampling using the bootstrap method.

Figure 8 shows the samples estimated by the proposed method of the frequency-weighted average angular velocity obtained from the *x*-axis gyro sensor at $\psi^{h}=90^{\circ }$ (${\bar{\omega }}_{x_{90}}^{c}$) together with the resampled samples by the bootstrap method. The upper and lower panels show box plots of the samples estimated by the proposed method and those resampled by the bootstrap method, respectively. The horizontal axis represents the angular velocity. The white circles show the outlier. The black dotted lines indicate the lower and upper limits of the two-sided 95% confidence interval for the population mean, calculated from the original sample using *t*-tests. The first and third quartiles of the bootstrap-resampled samples are within the two-sided 95% confidence interval calculated from the original sample. Therefore, the central 50% of the bootstrap-resampled values is contained within the confidence interval estimated from the original sample.

**Figure 9:**
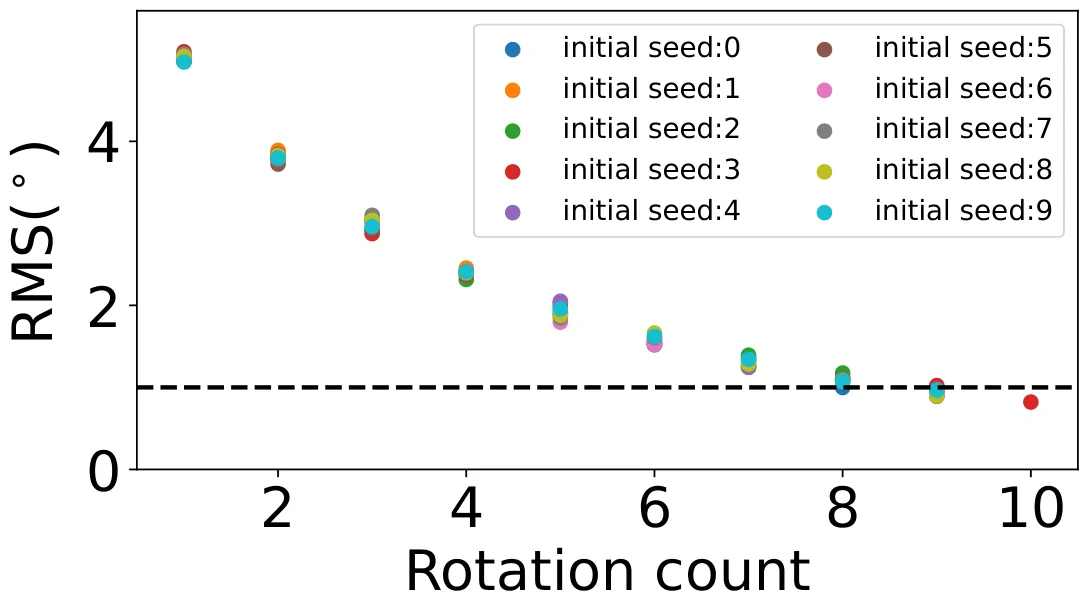
RMS errors of multiple lap trials.

The RMS errors of multiple lap trials are shown in Figure 9. In the figure, the vertical axis represents the RMS error and the horizontal axis represents the number of laps. The blue, orange, green, red, purple, brown, pink, gray, olive, and cyan dots show the result of initial seeds ranging from 0 to 9, respectively. The black dashed line shows the RMS = $1^{\circ }$. From this figure, it can be seen that the RMS error becomes less than $1^{\circ }$ within at most ten laps.

**Figure 10:**
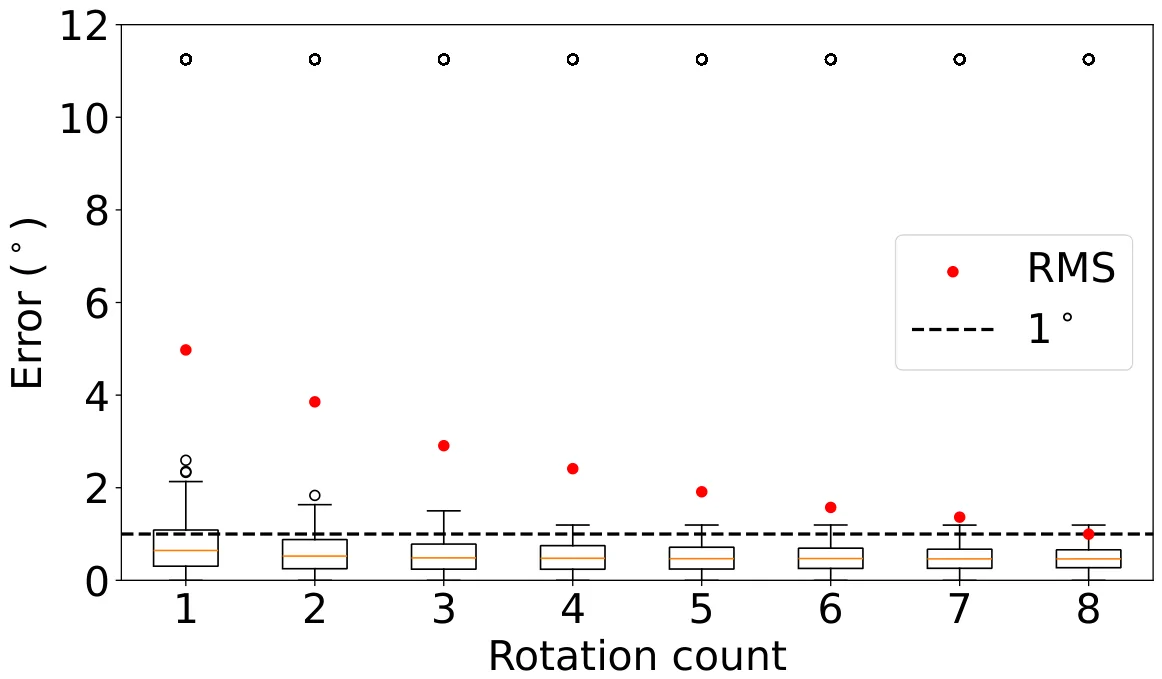
The distribution of azimuth estimation errors for each number of laps using an initial seed of 0.

The time required for the stepping motor to complete one lap is approximately 40 s, and since each measurement takes 25 s and 17 measurements are taken in all, the total time required for one lap is 465 s. Therefore, the proposed method achieved an RMS error of less than $1^{\circ }$ in approximately 1.3 h, demonstrating its effectiveness in such environments. This result shows a reduction in the required measurement time of approximately 2 h compared to our previous study [5], suggesting that the proposed method can contribute to practical operational systems. The distribution of the azimuth estimation errors for each number of laps using an initial seed of 0 is shown in Figure 10. The figure shows a box plot in which the vertical axis represents the error between the estimated phase and the theoretical value, with the horizontal axis representing the number of laps. The white circles show the outlier. In the figure, the red dots show the RMS value and the black dashed line shows the RMS = $1^{\circ }$. From this figure, it can be seen that although there are large outliers in each lap, the interval between quartiles narrows with each iteration and the median approaches 0.

## 6. Conclusions

This paper proposes a method for improving the initial azimuth estimation time of a north-finding system employing low-cost sensors and a compact 3-axis turntable. The system is capable of operating in non-horizontal and magnetically disturbed environments as well as environments where GNSS is unavailable. To verify the accuracy of the compact 3-axis turntable in the S-frame yaw ($\psi^{s}$), two types of verification were conducted. First, to verify rotation in the S-frame yaw ($\psi^{s}$), a rotation command of $22.5^{\circ }$ was continuously sent; images taken before rotation and after 100 laps were then compared using difference compositing. As a result, the alignment markers remained nearly unchanged in position, indicating that the compact 3-axis turntable achieves rotation in the S-frame yaw ($\psi^{s}$) that is consistent with the control signals. Second, a due north detection experiment was conducted. Data collected over 77 laps were resampled using a bootstrap method to construct a dataset of 5000 laps. Employing this dataset, multiple lap trials were conducted through 10,000 samplings with replacement, and the number of laps required to achieve an RMS error of less than 1° was investigated. The device was rotated in increments of $22.5^{\circ }$ to complete a lap starting from $\psi^{h}$ = 101.25° in order to verify the effectiveness of the proposed method under conditions in which due north was not known. The proposed method achieved an RMS error of less than $1^{\circ }$ in approximately 1.3 h, demonstrating its effectiveness in such environments. This result shows a reduction in the required measurement time of approximately 2 h compared to our previous study [5], suggesting that the proposed method can contribute to practical operational systems.

## Figures and Tables

**Table 1:** Parameters of the linear function of the offset error and scale error.

Element	Slope: *a*	Intercept: *b*	Element	Slope: *a*	Intercept: *b*
$\alpha_{x}^{c}$	$7.29\times 10^{-7}$	$-0.74\times 10^{-1}$	$\alpha_{x}^{r}$	$1.38\times 10^{-5}$	1.01
$\alpha_{y}^{c}$	$-4.56\times 10^{-5}$	$-0.18\times 10^{-1}$	$\alpha_{y}^{r}$	$1.60\times 10^{-5}$	1.02
$\alpha_{z}^{c}$	$-1.25\times 10^{-5}$	$-0.68\times 10^{-1}$	$\alpha_{z}^{r}$	$-3.39\times 10^{-6}$	1.00

**Figure A1:**
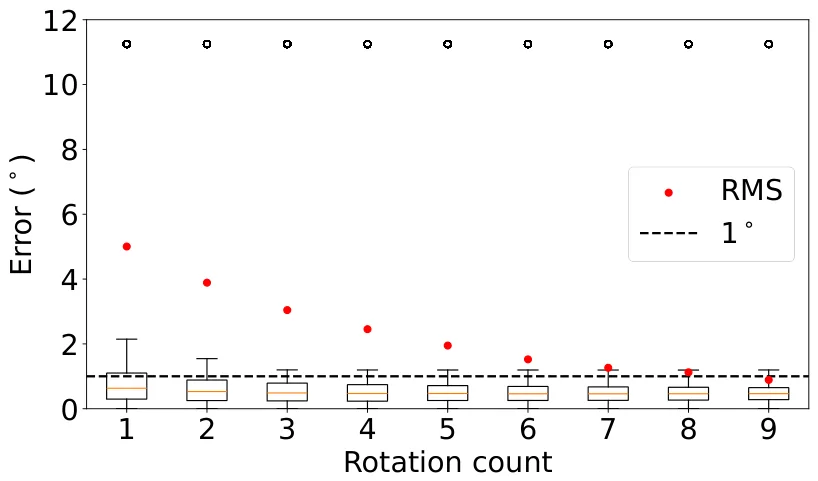
Distribution of azimuth estimation errors for each number of laps using an initial seed of 1.

**Figure A2:**
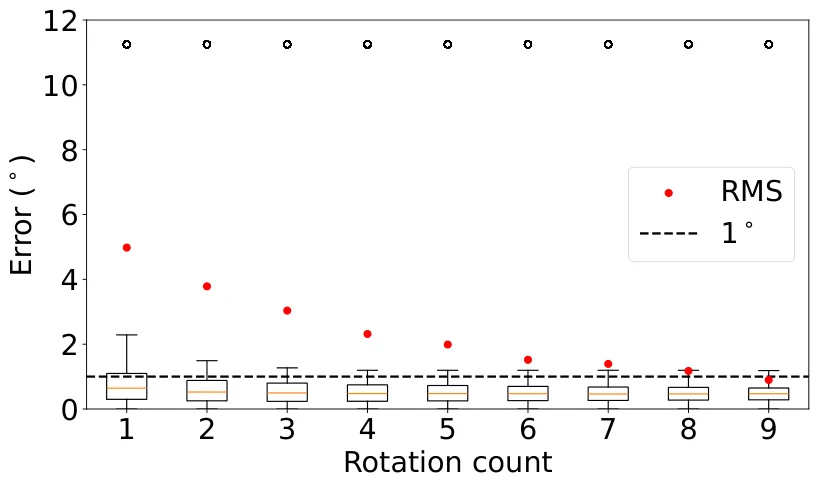
Distribution of azimuth estimation errors for each number of laps using an initial seed of 2.

**Figure A3:**
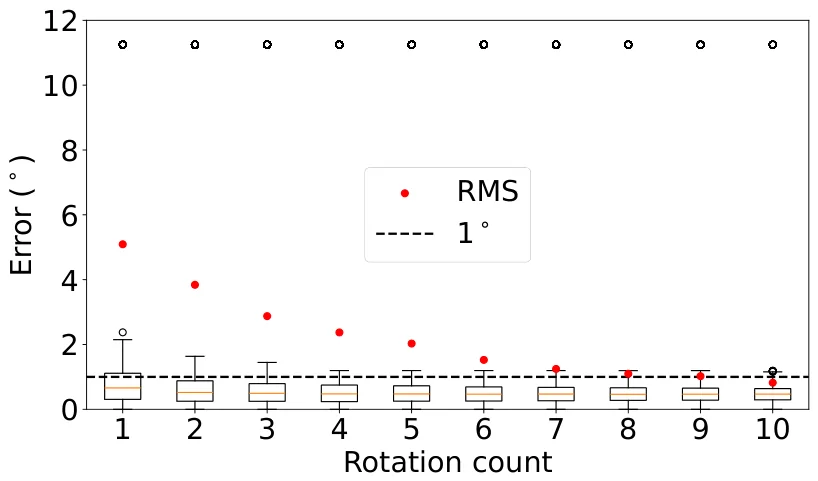
Distribution of azimuth estimation errors for each number of laps using an initial seed of 3.

**Figure A4:**
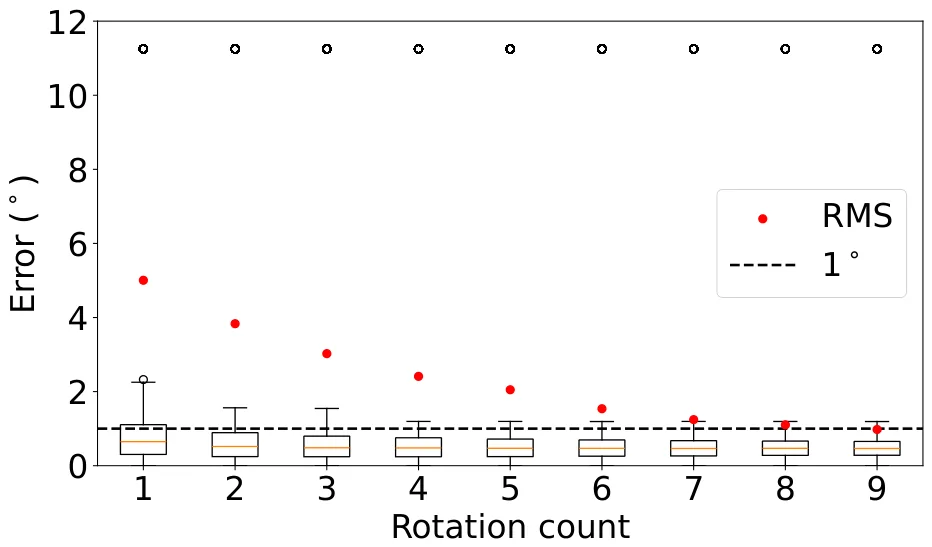
Distribution of azimuth estimation errors for each number of laps using an initial seed of 4.

**Figure A5:**
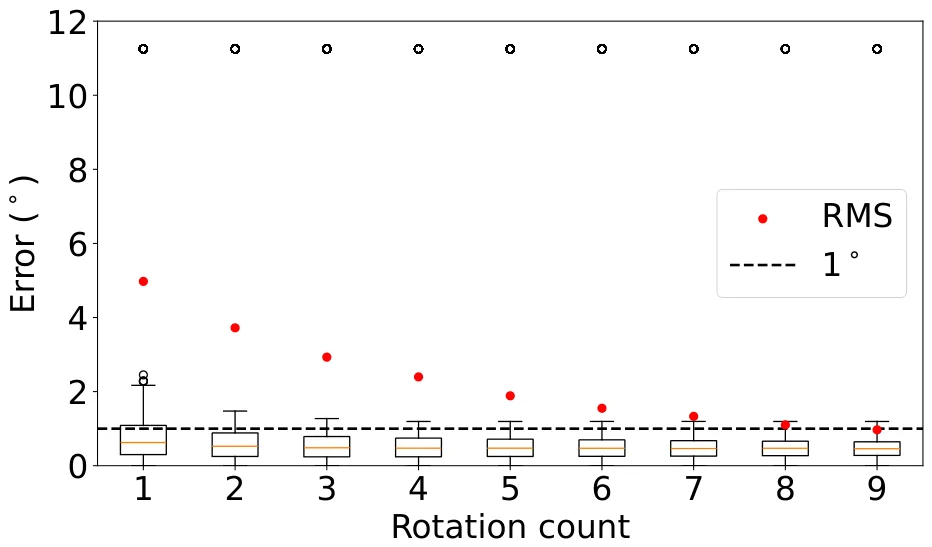
Distribution of azimuth estimation errors for each number of laps using an initial seed of 5.

**Figure A6:**
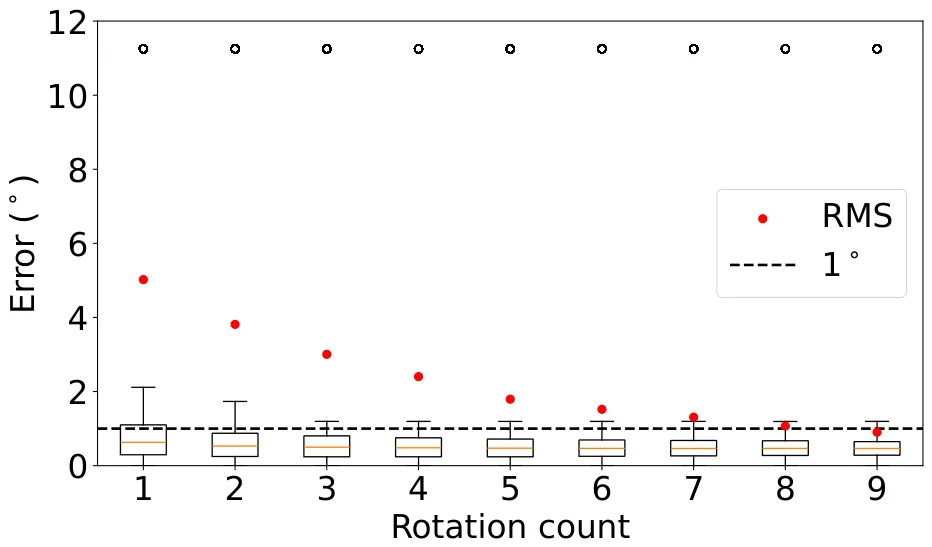
Distribution of azimuth estimation errors for each number of laps using an initial seed of 6.

**Figure A7:**
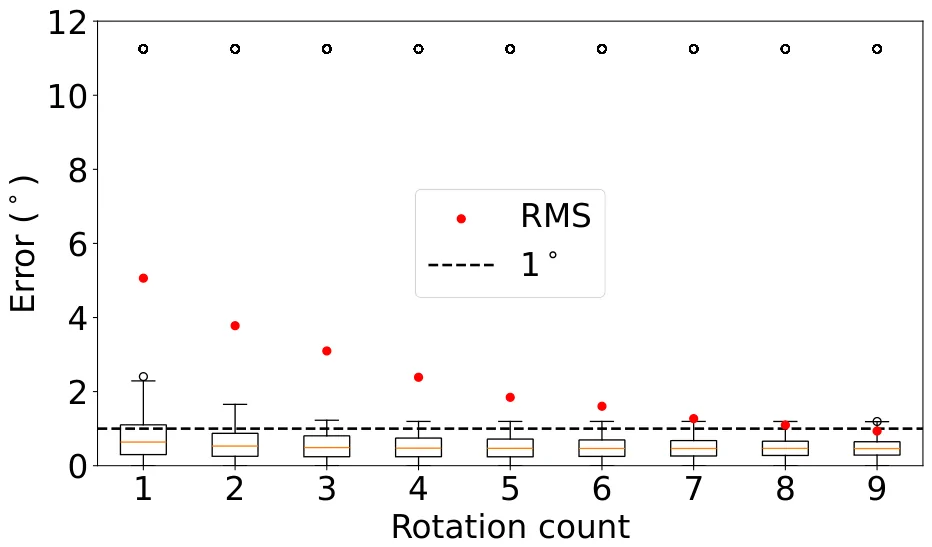
Distribution of azimuth estimation errors for each number of laps using an initial seed of 7.

**Figure A8:**
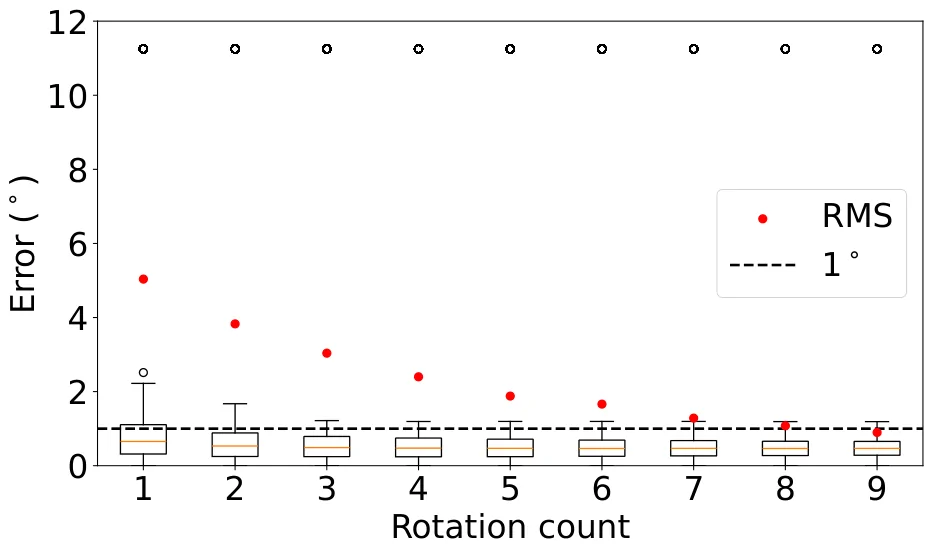
Distribution of azimuth estimation errors for each number of laps using an initial seed of 8.

**Figure A9:**
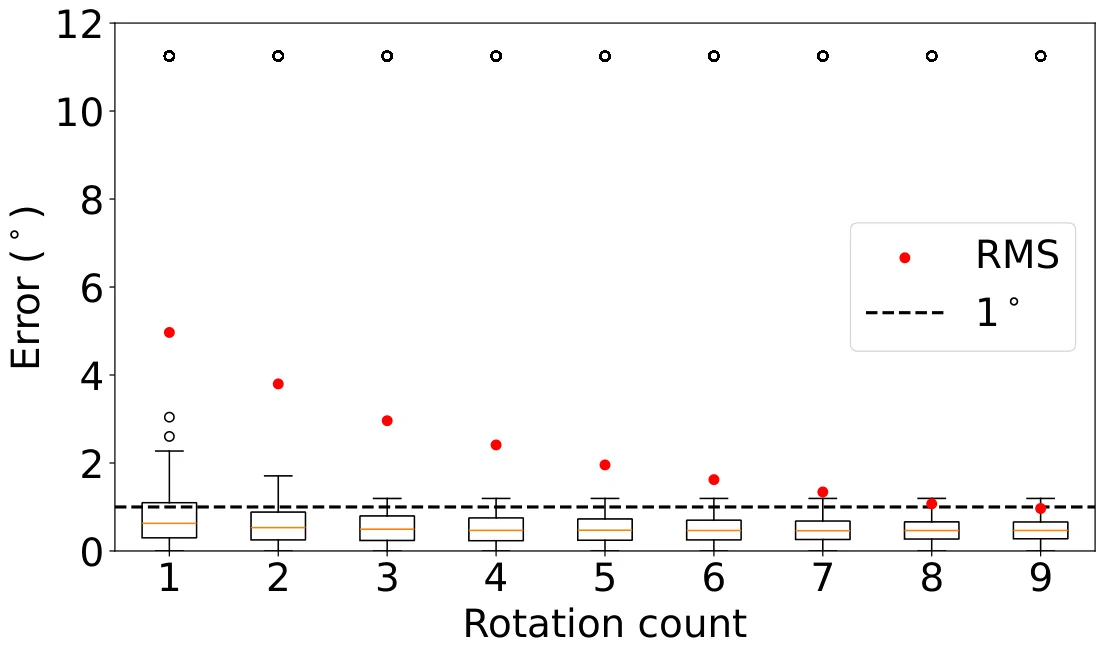
Distribution of azimuth estimation errors for each number of laps using an initial seed of 9.

## Data Availability

The raw data supporting the conclusions of this article will be made available by the authors on request.

## Abbreviations and Nomenclature

The following abbreviations are used in this manuscript:

S-frame	Sensor frame (the right-handed coordinate system with *z*-axis pointing downward)
C-frame	Computer frame (the rotation relative coordinate system)
MEMS	Micro Electro-Mechanical Systems
RKF	Robust Kalman filter
3D	Three-Dimensional
GNSS	Global Navigation Satellite System
RMS	Root Mean Square
$\alpha_{n}$	Acceleration $\left(m/s^{2}\right)$
$\omega_{n}$	Angular velocity ${(}^{\circ }/s)$
$\phi^{s}$	S-frame roll (rotation around the *x*-axis in the S-frame) ${(}^{\circ })$
$\theta^{s}$	S-frame pitch (rotation around the *y*-axis in the S-frame) ${(}^{\circ })$
$\psi^{s}$	S-frame yaw (rotation around the *z*-axis in the S-frame) ${(}^{\circ })$
$\phi^{c}$	C-frame roll (rotation around the *x*-axis in the C-frame) ${(}^{\circ })$
$\theta^{c}$	C-frame pitch (rotation around the *y*-axis in the C-frame) ${(}^{\circ })$
$\psi^{c}$	C-frame yaw (rotation around the *z*-axis in the C-frame) ${(}^{\circ })$
$\psi^{h}$	Heading angle in the C-frame ${(}^{\circ })$
Δt	Sampling interval (s)
g	Gravitational acceleration $\left(m/s^{2}\right)$
*	Quaternion product
⌈⌉	Ceil function
^⊤^	Transpose
∧	Logical AND

## Appendix A. Signal Processing

### Outlier Correction Utilizing the RKF [6]

The RKF is utilized to remove outliers in the acceleration $\alpha_{n}$, angular velocity $\omega_{n}$, and angular velocity estimated from the acceleration $\omega_{n}^{\alpha }$. For outlier correction of acceleration, let the state be x and the observation y. Their state–space model is represented by the following Equation (A1).

(A1) $$\begin{array}{c} \left\{\begin{array}{c} x_{n}=x_{n-1}+v_{n}\\ y_{n}=x_{n}+w_{n}+z_{n} \end{array}\right. \end{array}$$

The state x and observation y for each subject are shown in Table A1.

**Table A1:** State x and observation y.

Item	*x*	*y*
ω	${\left[{\hat{\omega }}_{x},{\hat{\omega }}_{y}\right]}^{⊤}$	${\left[\omega_{x},\omega_{y}\right]}^{⊤}$
α	${\left[{\hat{\alpha }}_{x},{\hat{\alpha }}_{y},{\hat{\alpha }}_{z}\right]}^{⊤}$	${\left[\alpha_{x},\alpha_{y},\alpha_{z}\right]}^{⊤}$
$\omega^{\alpha }$	${\left[{\hat{\omega }}_{x_{n}}^{\alpha },{\hat{\omega }}_{y_{n}}^{\alpha }\right]}^{⊤}$	${\left[\omega_{x_{n}}^{\alpha },\omega_{y_{n}}^{\alpha }\right]}^{⊤}$

In Equation (A1), $v_{n}$ is the system noise vector and is the Gaussian white noise sequence N(0,Q); $w_{n}$ is the observation noise vector and is the Gaussian white noise sequence N(0,R); $z_{n}$ is the outlier vector; and R is the variance–covariance matrix of the observation noise. The variance–covariance matrices of the system noise Q and observation noise R for each subject are shown in Table A2. The variance–covariance matrix parameters for each subject are shown in Table A3. For simplicity, in this study the variance–covariance matrix of the system noise Q is approximated as a scalar multiple of a diagonal covariance matrix. Note that the state vector x, observation vector y, and outlier vector z should not be confused with the coordinate-system *x*-, *y*-, and *z*-axes.

**Table A2:** Variance–covariance matrices of the system noise Q and observation noise R.

Item	*Q*	*R*
ω	$0.001\mathrm{diag}(\sigma_{x}^{2},\sigma_{y}^{2})$	$\left[\begin{array}{cc} \sigma_{x}^{2} & \sigma_{xy}\\ \sigma_{xy} & \sigma_{y}^{2} \end{array}\right]$
α	$\mathrm{diag}(\sigma_{x}^{2},\sigma_{y}^{2},\sigma_{z}^{2})$	$\left[\begin{array}{ccc} \sigma_{x}^{2} & \sigma_{xy} & \sigma_{xz}\\ \sigma_{xy} & \sigma_{y}^{2} & \sigma_{yz}\\ \sigma_{xz} & \sigma_{yz} & \sigma_{z}^{2} \end{array}\right]$
$\omega^{\alpha }$	$0.01\mathrm{diag}(\sigma_{x}^{2},\sigma_{y}^{2})$	$\left[\begin{array}{cc} \sigma_{x}^{2} & \sigma_{xy}\\ \sigma_{xy} & \sigma_{y}^{2} \end{array}\right]$

**Table A3:** The variance–covariance matrix parameters used in the RKF.

Item	$\sigma_{x}$	$\sigma_{y}$	$\sigma_{z}$	$\sigma_{xy}$	$\sigma_{xz}$	$\sigma_{yz}$
ω	$0.25\times 10^{-2}$	$0.23\times 10^{-2}$	–	$-0.11\times 10^{-6}$	–	–
α	$0.77\times 10^{-2}$	$0.71\times 10^{-3}$	$0.21\times 10^{-3}$	$-0.12\times 10^{-4}$	$-0.18\times 10^{-1}$	$0.11\times 10^{-1}$
$\omega^{\alpha }$	$0.25\times 10^{-2}$	$0.23\times 10^{-2}$	–	$-0.11\times 10^{-6}$	–	–

The state estimation of Equation (A1) can be carried out utilizing the RKF algorithm shown in Equations (A2)–(A9).

[One-step-ahead prediction]

(A2) $$\begin{array}{cc} \; & {\hat{x}}_{n|n-1}={\hat{x}}_{n-1|n-1} \end{array}$$

(A3) $$P_{n|n-1}=P_{n-1|n-1}+Q$$

[Filtering]

(A4) $$\begin{array}{cc} \; & \Sigma_{n}=P_{n|n-1}+R \end{array}$$

(A5) $$\begin{array}{cc} \; & L=P_{n|n-1}{\left(\Sigma_{n}\right)}^{-1} \end{array}$$

(A6) $$\begin{array}{cc} \; & e_{n}=y_{n}-{\hat{x}}_{n|n-1} \end{array}$$

(A7) $$\begin{array}{cc} \; & {\hat{z}}_{k,n}=\left\{\begin{array}{cc} e_{k_{n}}-\sqrt{\Sigma_{kk_{n}}} & \mathrm{if}\;e_{k_{n}}\geq \sqrt{\Sigma_{kk_{n}}}\\ e_{k_{n}}+\sqrt{\Sigma_{kk_{n}}} & \mathrm{if}\;e_{k_{n}}<-\sqrt{\Sigma_{kk_{n}}}\\ 0 & \mathrm{otherwise} \end{array}\right.\left(k\in \left\{x,y,z\right\}\right) \end{array}$$

(A8) $$\begin{array}{cc} \; & {\hat{x}}_{n|n}={\hat{x}}_{n|n-1}+L\left(e_{n}-{\hat{z}}_{n}\right) \end{array}$$

(A9) $$\begin{array}{cc} \; & P_{n|n}=P_{n|n-1}-LP_{n|n-1} \end{array}$$

where P is the variance–covariance matrix of $\hat{x}$ and its initial value is set equal to the variance–covariance matrix of the system noise Q.

## Appendix B. Experimental Results

The experimental results are shown in Figure A1, Figure A2, Figure A3, Figure A4, Figure A5, Figure A6, Figure A7, Figure A8 and Figure A9. The figures show a box plot in which the vertical axis represents the error between the estimated phase and theoretical value, while the horizontal axis represents the number of laps. In the figure, the red dots show the RMS value and the black dashed line shows the RMS = $1^{\circ }$.
